# Genetically engineered *Pseudomonas aeruginosa* with lipase regulation for production of rhamnolipids from waste frying oil

**DOI:** 10.3389/fmicb.2025.1691217

**Published:** 2025-11-20

**Authors:** Runyu Yang, Yitong Shi, Jinyu Bian, Mengxiao Sun, Junchi Ma, Cailing Ren, Xueyan Feng, Yun Wang, Yuanhui Yang, Jianqiang Zhou, Jun Fu, Ruijuan Li

**Affiliations:** 1 State Key Laboratory of Microbial Technology, Shandong University, Qingdao, China; 2 JiaBioSyn (Shanghai) Biotechnology Co., Ltd, Shanghai, China; 3 Shanghai Wenxin Biotechnology Co., Ltd., Shanghai, China

**Keywords:** *Pseudomonas aeruginosa*, waste frying oil, rhamnolipids, genetic engineering, lipase, fermentation

## Abstract

**Introduction:**

Rhamnolipids are valuable biosurfactants, but their large-scale application is limited by high production costs. Waste frying oil (WFO), a low-cost and abundant triglyceride-rich byproduct, offers a sustainable carbon source for rhamnolipid synthesis, though its utilization efficiency by microbes like *Pseudomonas aeruginosa* needs improvement.

**Methods:**

This study evaluated the potential of engineered *P. aeruginosa* PAO1 strains (wild-type PAO1, aroA knockout PAO1ΔaroA, RhlAB/estA-overexpressing PAO1-RhlAB, lipase-overexpressing PAO1-lipase) for rhamnolipid production using WFO as the sole carbon source (soybean oil as positive control). Strategies to enhance WFO utilization—endogenous lipase overexpression and exogenous lipase addition (PAO1+, PAO1-lipase+ with repeated supplementation)—were also tested.

**Results:**

Wild-type PAO1 and PAO1ΔaroA could synthesize rhamnolipids from WFO, while PAO1-RhlAB improved yields vs. PAO1 in WFO. Exogenous lipase addition (PAO1+) achieved 14.0 g/L rhamnolipids at 48 h (vs. 9 g/L for PAO1), and the synergistic PAO1-lipase+ reached 16.0 g/L (vs. 12.8 g/L for PAO1-lipase) at the same time. At 144 h, PAO1-lipase+ had the highest oil degradation rate (34.40%), while PAO1+ maintained a slightly higher yield (20 g/L) than PAO1-lipase+ (19 g/L).

**Discussion:**

These findings confirm that lipase regulation (overexpression or exogenous addition) enhances WFO utilization and rhamnolipid production, providing a cost-effective approach for sustainable rhamnolipid biosynthesis from waste lipids.

## 1 Introduction

Rhamnolipids, a class of anionic glycolipid biosurfactants, have garnered significant attention due to their remarkable properties and diverse applications (Chen et al., 2017; Thakur et al., 2021; Kabeil et al., 2025). These biosurfactants are composed of one or two rhamnose sugar units linked to one or two *β*-hydroxy fatty acid chains (Wittgens and Rosenau, 2018). The unique amphiphilic structure of rhamnolipids endows them with excellent surface-active properties, such as the ability to reduce surface and interfacial tensions, emulsify hydrophobic substances, and form stable micelles (Tiso et al., 2017; Jahan et al., 2020). Structurally, the relationship between rhamnolipid composition and activity remains a focus. Recent studies have shown that subtle variations in the length of the fatty acid chains and the number of rhamnose units can significantly alter their surface activity and biological functions (Nasir et al., 2025). For example, longer fatty acid chains enhance emulsifying capabilities, while multiple rhamnose units increase antibacterial activities (Das et al., 2014; Li et al., 2022). Advanced analytical techniques, such as nuclear magnetic resonance (NMR) spectroscopy and mass spectrometry (MS), enable precise structural characterization underpinning further exploration of their properties and applications (Behrens et al., 2016; Marshall and Powers, 2017).

Rhamnolipids exhibit diverse applications across multiple industries. In the petroleum industry, rhamnolipids contribute to enhanced oil recovery (EOR) by two key mechanisms: first, reducing the oil–water interfacial tension to mobilize trapped oil, and second, solubilizing or emulsifying heavy oils (Chen and Lee, 2022; Kashif et al., 2022). However, high production costs limit their commercial viability against synthetic surfactants (de Oliveira Schmidt et al., 2021). In environmental remediation, rhamnolipids effectively bioremediate hydrocarbons, heavy metals, and other organic pollutants (Liu et al., 2018). They enhance pollutant bioavailability for microbial degradation (Mishra et al., 2021). For instance, in the treatment of soil contaminated with polycyclic aromatic hydrocarbons (PAHs), rhamnolipids can form complexes with PAHs, increasing solubility and bacteria access. Additionally, rhamnolipids have shown the ability to chelate heavy metals, reducing their toxicity and mobility in the environment (Parus et al., 2023). Nevertheless, their stability in complex environmental matrices requires further investigation. In the food industry, rhamnolipids are considered as potential alternatives to synthetic surfactants due to their low toxicity and biodegradability (Soares Dos Santos et al., 2016; Liu et al., 2018; Thakur et al., 2021). Studies have reported that rhamnolipids can effectively inhibit the growth of foodborne pathogens, such as *Listeria monocytogenes* and *Salmonella enterica*, without causing harm to human health (Chlumsky et al., 2021). In the pharmaceutical and medical fields, rhamnolipids show antibacterial, antifungal, and antiviral activities (Da Silva et al., 2020; Giugliano et al., 2021; Buonocore et al., 2023). Their biofilm-disrupting capability has attracted significant interest in the treatment of chronic infections (Soberon-Chavez et al., 2011) and potentiating antibiotics by interfering with bacterial adhesion and communication (Nickzad and Deziel, 2014). However, the development of rhamnolipid-based pharmaceuticals is still in its early stages, and issues such as large-scale production, purification, and formulation need to be resolved.

*Pseudomonas aeruginosa* is the most common and efficient producers of rhamnolipids (Soberon-Chavez et al., 2005; Reis et al., 2011), utilizing a wide range of carbon sources including sugars, organic acids, and hydrocarbons (Pathania and Jana, 2020; Li et al., 2024). The ability to adapt to different carbon sources provides flexibility in the production of rhamnolipids and allows for the exploration of cost-effective raw materials (Pathania and Jana, 2020). Notably, the lipase produced by *P. aeruginosa*, especially the model strain PAO1, exhibits efficient secretory capacity—this characteristic enables the enzyme to be readily released into the extracellular environment, which is highly beneficial for improving the utilization efficiency of extracellular lipids. By acting directly on triglycerides at the oil–water interface without relying on intracellular uptake of large lipid molecules, the secreted lipase can more efficiently drive the hydrolysis of extracellular lipids, laying a critical foundation for subsequent rhamnolipid synthesis. The metabolic pathways of rhamnolipid biosynthesis in *P. aeruginosa* have been relatively well-studied (Zheng et al., 2023). The *rhlAB* operon encodes enzymes responsible for the synthesis of the basic rhamnolipid structure, while the *rhlCDE* operon is involved in the regulation and modification of rhamnolipid production (Wittgens et al., 2017). However, the regulation of these pathways is complex, involving multiple regulatory factors and environmental signals, such as quorum sensing systems and nutrient availability (Reis et al., 2011). In recent years, gene editing technologies have emerged as powerful tools to dissect and engineer regulatory networks in *P. aeruginosa* (Li et al., 2023; Yin et al., 2019). For instance, CRISPR-Cas3 systems have successfully knocked out or overexpressed key regulatory genes (Zheng et al., 2022, 2023), enhancing rhamnolipid yields by alleviating metabolic bottlenecks. Precise understanding and manipulating of these regulatory mechanisms are essential for rhamnolipid production optimization. Moreover, targeted gene editing of the *rhlAB* and *rhlCDE* operons themselves has enabled fine-tuning of enzyme expression levels, improving substrate conversion efficiency and yielding rhamnolipid variants with enhanced surface-active properties.

High production cost hinders large-scale application (Haba et al., 2000). Waste frying oil (WFO), a triglyceride-rich food industry byproduct, offers a sustainable, low-cost carbon source. *Pseudomonas aeruginosa* lipases hydrolyze triglycerides into fatty acids and glycerol for rhamnolipid synthesis (Nasir et al., 2025). However, WFO utilization faces challenges: variable composition (due to origin and frying conditions) and rate-limiting triglyceride hydrolysis step, with wild-type lipase efficiency insufficient for high yields, especially in oxidized or complex WFO matrices (Elazzazy et al., 2015). To address these challenges, our study focuses on two complementary strategies: (i) Genetic engineering of *rhlAB* (core biosynthetic operon) and *estA* (a triglyceride-hydrolyzing esterase) (Elazzazy et al., 2015; Nasir et al., 2025) to enhance metabolic flux toward rhamnolipid synthesis; (ii) Modulating lipase, a key enzyme in triglyceride hydrolysis; through three lipase strategies: (i) endogenous overexpression (PAO1-lipase), (ii) exogenous supplementation (PAO1+), and (iii) their synergistic combination (PAO1-lipase+). These accelerate triglyceride hydrolysis, overcome WFO recalcitrance, and shorten fermentation cycles caused by delayed rhamnolipid synthesis (typically post-critical cell density). By integrating *rhlAB*/*estA* engineering with lipase regulation, this study enhances rhamnolipid yields from WFO, validates its industrial viability, and provides a sustainable, cost-effective biosurfactant production route.

## 2 Materials and methods

### 2.1 Strains, plasmids, and growth conditions

The strains and plasmids used in this study are listed in Supplementary Table 1. *Escherichia coli* and *P. aeruginosa* were cultured in LB medium (tryptone, 10 g/L; yeast extract, 5 g/L; NaCl, 1 g/L; agar for solid medium, 15 g/L). The fermentation seed culture was prepared using LB medium with 10 g/L NaCl. Antibiotics were supplemented when necessary: 300 μg/mL kanamycin, 15 μg/mL gentamicin.

### 2.2 Fermentation conditions

Add the seed solution to the fermentation medium at a 10% dilution rate. Fermentation medium: Using 125 g/L soybean oil or 125 g/L waste frying oil as the sole carbon source, the mineral-free solution contains 15.0 g/L NaNO₃, 0.5 g/L MgSO₄·7H₂O, 1.0 g/L KCl, and 0.3 g/L K₂HPO₄ as the phosphorus source. The pH is adjusted to 6.5 and controlled during the cultivation process. During the cultivation period, 1 mL/L of trace element solution is added at 0, 20, 40, 70, and 120 h to provide a stable and suitable nutritional environment for microbial growth and rhamnolipid synthesis. Add three aromatic amino acids, phenylalanine, tryptophan, and tyrosine (50 μg/mL), to the *aroA* mutant strain fermentation medium; add 1 mL/L of trace element solution at 0 h, 20 h, 40 h, 70 h, and 120 h, respectively.

Trace element composition (filtered through a 0.22 μm filter membrane): 2.0 g/L sodium citrate × 2H₂O (citric acid sodium), 0.28 g/L FeCl₃ × 6H₂O, 1.4 g/L ZnSO₄ × 7H₂O, 1.2 g/L CoCl₂ × 6H₂O, 1.2 g/L CuSO₄ × 5H₂O, 0.8 g/L MnSO₄ × H₂O. The waste oils are all treated waste oils, and they come from the canteens of Shanghai and the Qingdao campus of Shandong University.

WFO primarily consists of triglycerides (with saturated/unsaturated fatty acids), along with free fatty acids from hydrolysis, hydroperoxides and aldehydes/ketones from oxidation, polymerized triglycerides from polymerization, plus impurities like food residues and moisture. To standardize WFO quality before fermentation, all waste oil samples were processed by centrifugation at 10,000×*g* for 20 min to remove food debris and water. Acid value was measured using KH titration method, with results expressed as mg KOH per gram of oil.

### 2.3 Electroporation method of *P. aeruginosa*

Pick a single colony from the plate and incubate it at 37 °C for overnight. Then, transfer an appropriate amount of seed solution to fresh bacterial solution to make the initial OD_600_ 0.1. Incubate at 37 °C for 2 h. After reaching OD_600_ of 0.6–0.8, wash twice with ddH_2_O, add the target plasmid at 500 ng, mix well, and transfer to a 1 mm electroporation cup. Use an Eppendorf electroporation instrument at 1,300 V for electroporation. After removal, add 1 mL of antibiotic-free LB medium, and incubate at 37 °C for 1 h before plating onto the corresponding antibiotic plate.

### 2.4 Extracting rhamnolipids and detecting them using the anthrone method

During the fermentation process, regular sampling was conducted at specific time points—either at 48 h, 72 h, 96 h, 120 h or at 48 h, 96 h, 120 h, 144 h. After the fermentation broth was allowed to stand (to facilitate phase separation), 2 mL of the aqueous phase was collected into an EP tube for subsequent analysis The samples were mixed with petroleum ether at a ratio of 1:1 (v/v), and then centrifuged at 4,618 g and 4 °C for 30 min to separate the biomass, aqueous phase and organic phase. The organic phase was evaporated to dryness with n-hexane and used for oil content determination. The pH of the aqueous phase was adjusted to 2–3, and then extracted twice with ethyl acetate at a ratio of 1:1 (v/v) for rhamnolipid identification and HPLC quantitative analysis.

For the detection of rhamnolipids using the anthrone method, the reagents were prepared as follows: 0.2 g of anthrone was dissolved in 100 mL of concentrated sulfuric acid to prepare the anthrone reagent, which was freshly made on the day of use; a 1 mg/mL rhamnolipid stock solution was prepared with distilled water, and then serially diluted to obtain standard solutions of 0.1 mg/mL, 0.2 mg/mL, 0.3 mg/mL, 0.4 mg/mL, and 0.5 mg/mL. For constructing the standard curve, 1.0 mL of each standard solution was added to stoppered graduated test tubes (3 replicates per concentration), followed by the accurate addition of 4.0 mL of anthrone reagent, which was immediately and thoroughly mixed (Thakur et al., 2021). The tubes were then heated in a boiling water bath for 10 min (with precautions against splashing), transferred to an ice bath to cool to room temperature (~10 min), and their absorbance was measured at 620 nm using a visible spectrophotometer with a blank control (1.0 mL distilled water + 4.0 mL anthrone reagent). Finally, a standard curve was plotted with rhamnolipid concentration as the abscissa and absorbance as the ordinate, and the regression equation was calculated.

### 2.5 HPLC and MS analysis

For sample pretreatment, 10 mL of fermentation broth is centrifuged at 8,000 rpm for 10 min at 4 °C. The pH of the supernatant is adjusted to 2.0 with 1 M hydrochloric acid, and 12.5 mL of ethyl acetate (v/v 1:1.25) is added for shaking extraction for 10 min. After centrifugation at 12,000 rpm for 5 min, the organic phase is collected. The extraction is repeated once (resulting in a total of two extractions), and the combined organic phases are dried by nitrogen blowing and redissolved with 1 mL of methanol, followed by filtration through a 0.22 μm filter membrane for later use. Chromatographic analysis is performed on a C18 reversed-phase chromatographic column (250 mm × 4.6 mm, 5 μm) with a mobile phase of acetonitrile-water (volume ratio 60:40) containing 0.1% trifluoroacetic acid, a flow rate of 1.0 mL/min, a column temperature of 30 °C, an injection volume of 10 μL, and a detection wavelength of 210 nm. Mass spectrometry uses an electrospray ionization (ESI) source in positive ion mode, with a scanning range of *m*/*z* 100–1,000, a capillary voltage of 3.0 kV, a cone voltage of 50 V, an ion source temperature of 150 °C, and a desolvation gas temperature of 350 °C with a flow rate of 700 L/h (Rudden et al., 2015). The structure of rhamnolipid homologs is confirmed by detecting the [M + H]^+^ positive ion peaks (dirhamnolipid *m*/*z* about 651, monorhamnolipid *m*/*z* about 505) and characteristic fragment ions, combined with the comparison of standard mass spectra (Supplementary Figures 1–3).

### 2.6 The method for determining glycerol content

Centrifuge the fermentation supernatant at 12,000 rpm for 2 min. Then, perform glycerol measurement using the Amplex Red Glycerol Assay Kit (S0223S) of Biyuntian Company according to the manufacturer’s instructions. Add 20 μL of fermentation supernatant to the sample wells of a 96-well plate, and set up blank controls simultaneously. Add 80 μL of glycerol detection working solution to each well, mix well, and incubate at 37 °C in the dark for 30 min. Then, detect using a microplate reader at 570 nm (A570).

## 3 Results and discussion

### 3.1 RhlAB overexpression enables rhamnolipid production using lipids as the sole carbon source in PAO1

**Figure 1:**
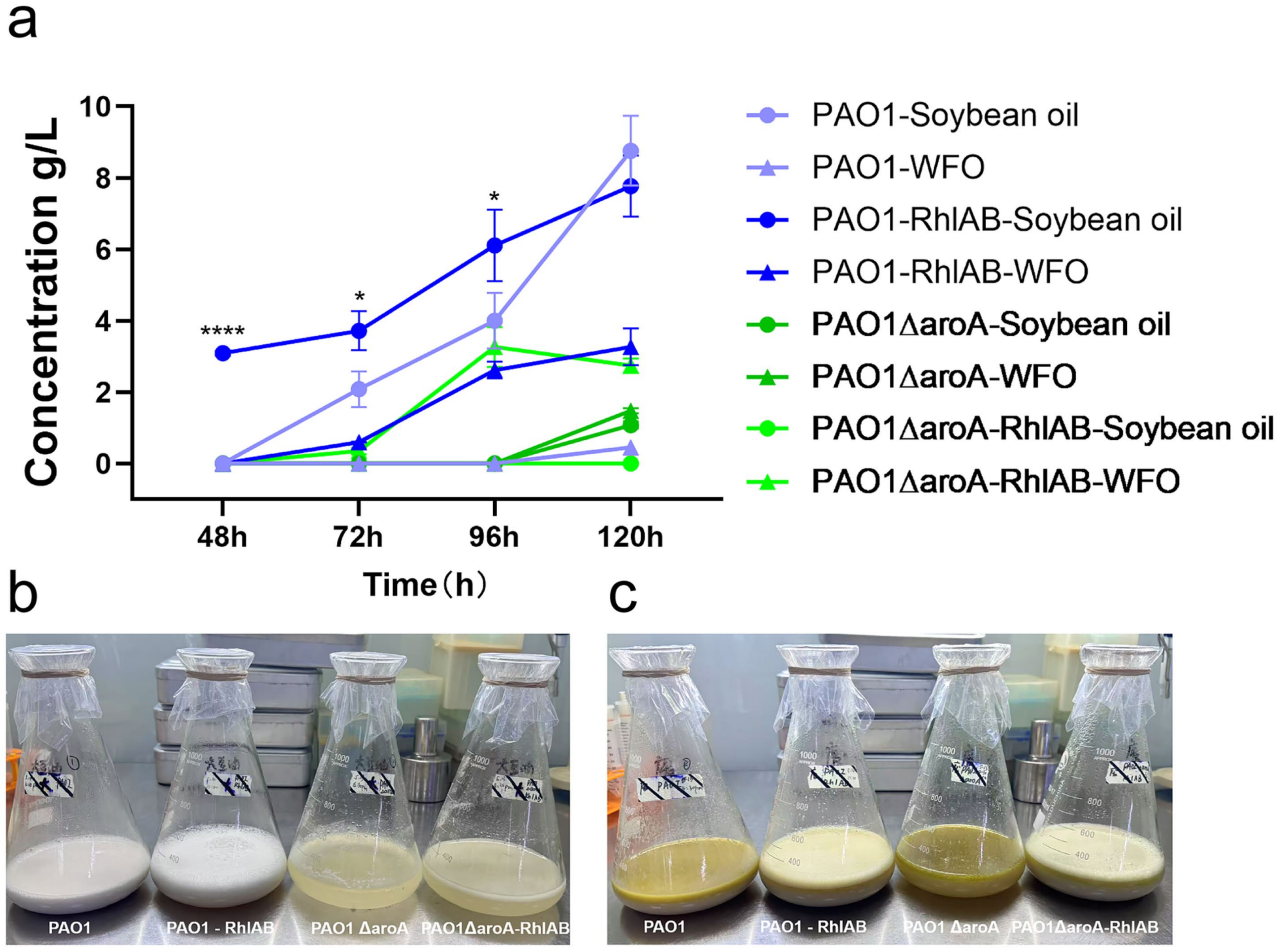
Rhamnolipid production by *P. aeruginosa* strains using soybean oil or WFO as the sole carbon source. **(a)** Time-course analysis of rhamnolipid concentration (g/L) for four strains—PAO1, PAO1-RhlAB (RhlAB-overexpressing), PAO1ΔaroA (*aroA* knockout), and PAO1ΔaroA-RhlAB (*aroA* knockout with RhlAB overexpression)—cultured in soybean oil or WFO over 120 h. Data are presented as means ± standard deviation (*n* = 3). **(b)** Visual comparison of fermentation broths at 120 h for strains cultured in soybean oil. From left to right: PAO1, PAO1-RhlAB, PAO1ΔaroA, PAO1ΔaroA-RhlAB. **(c)** Visual comparison of fermentation broths at 120 h for strains cultured in WFO. From left to right: PAO1, PAO1-RhlAB, PAO1ΔaroA, PAO1ΔaroA-RhlAB.

To assess lipid substrate utilization and RhlAB overexpression effects, fermentation experiments were conducted with soybean oil (positive control) and WFO as the sole carbon sources, using wild-type PAO1, PAO1-RhlAB (RhlAB-overexpressing strain), PAO1ΔaroA (*aroA* knockout strain), and PAO1ΔaroA-RhlAB (*aroA* knockout with RhlAB overexpression strain). Rhamnolipid yields were monitored at 48 h, 72 h, 96 h, and 120 h (Figure 1).

Rhamnolipid concentrations increased with fermentation time in most strains. Notably, the PAO1-RhlAB strain exhibited significantly higher rhamnolipid production compared to the wild-type PAO1 strain during the early fermentation stages (48 h *p* < 0.0001, 72 h *p* = 0.0178, and 96 h *p* = 0.0445) in soybean oil. At 96 h, PAO1-RhlAB reached ~6 g/L rhamnolipid, which was higher than that of PAO1 (around 4 g/L). However, at 120 h, PAO1 surpassed PAO1-RhlAB, achieving ~9 g/L versus ~8 g/L, though this difference was not statistically significant (*p* = 0.2573). This observed shift in production dynamics may stem from several potential factors. Firstly, RhlAB overexpression could potentially impose a metabolic burden on the cells that intensifies during fermentation, which might divert cellular resources from rhamnolipid synthesis. Secondly, the regulatory mechanisms of rhamnolipid biosynthesis in *P. aeruginosa* are complex, and overexpression of RhlAB alone may not be sufficient to sustain enhanced production throughout the entire fermentation period, as other genes or pathways could potentially become rate-limiting. Additionally, wild-type PAO1 might have a more balanced metabolic regulation that could allow for sustained rhamnolipid synthesis during later fermentation stages. In future work, we plan to construct engineered chassis strains through systematic modification of the global metabolic flux in bacteria, thereby enabling overexpressing strains to achieve more robust improvements in rhamnolipid production.

In WFO cultures, PAO1-RhlAB consistently outperformed PAO1 throughout the fermentation period, reaching ~4 g/L at 120 h versus ~1 g/L. This suggests that RhlAB overexpression is particularly beneficial when the carbon source is less favorable, such as WFO, which contains oxidation products and other impurities that could hinder bacterial metabolism. This observation aligns with prior studies demonstrating that WFO, despite its impurities, can serve as an effective low-cost carbon source for rhamnolipid production, and that genetic modification of key biosynthetic genes enhances adaptability to such suboptimal substrates (Sun et al., 2021). Notably, RhlAB encodes the rhamnosyltransferase 1 complex, a rate-limiting enzyme in rhamnolipid biosynthesis, and its overexpression is a well-documented strategy to boost yields, especially in systems where precursor availability or enzyme activity may be constrained by substrate quality (Liu et al., 2025). In contrast, the PAO1ΔaroA strain showed relatively low rhamnolipid production in both carbon sources, confirming *aroA* knockout’s negative impact. However, combining RhlAB overexpression (PAO1ΔaroA-RhlAB) partially restored yields, especially in WFO. At 120 h, PAO1ΔaroA-RhlAB reached a rhamnolipid concentration of ~ 3 g/L in WFO, which was significantly higher than that of PAO1ΔaroA (~1 g/L). Thus, while *aroA* knockout impairs rhamnolipid production, RhlAB overexpression can partially mitigate this effect.

### 3.2 Waste oil origin influences rhamnolipid yield

**Figure 2:**
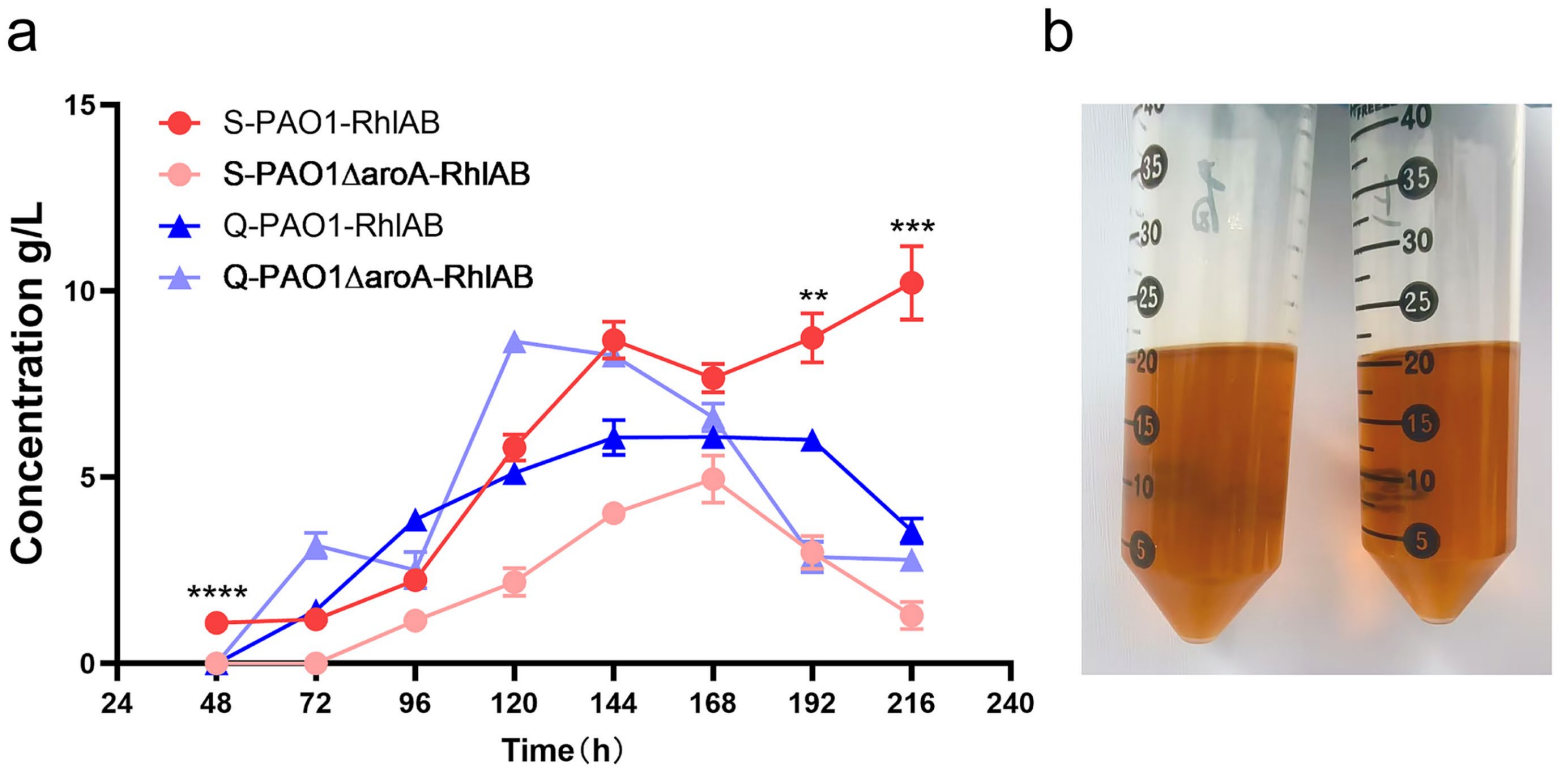
Impact of WFO source on rhamnolipid production by *P. aeruginosa* strains. **(a)** Time-course analysis of rhamnolipid concentration (g/L) for two strains—PAO1-RhlAB (RhlAB-overexpressing) and PAO1ΔaroA-RhlAB (*aroA* knockout with RhlAB overexpression)—cultured in Shanghai-derived WFO (S) or Qingdao-derived WFO (Q) over 216 h. Data are presented as means ± standard deviation (*n* = 3). **(b)** Visual comparison of Qingdao-derived WFO solubility and lipid content. Left: Qingdao WFO diluted 1:1 in petroleum, right: Shanghai WFO diluted 1:1 in petroleum, confirming lipid content of ≥99% (clear, homogeneous oil phase).

We compared Shanghai (S) and Qingdao (Q) WFOs using *P. aeruginosa* strains PAO1-RhlAB (RhlAB-overexpressing) and PAO1ΔaroA-RhlAB (*aroA* knockout with RhlAB overexpression). Both WFO samples had a lipid content of ≥99%, but differed significantly in acid value (S: 69 mg KOH/g; Q: 520 mg KOH/g). Rhamnolipid yields were monitored over 216 h (Figure 2).

Results showed that the geographical origin of WFO strongly influenced rhamnolipid production. The S-WFO group (red and pink lines) consistently outperformed the Q-WFO group (blue and purple lines) across all time points. For instance, S-PAO1-RhlAB reached a maximum yield of 10 g/L at 216 h, while Q-PAO1-RhlAB only achieved 4 g/L at the same time. Similarly, S-PAO1ΔaroA-RhlAB reached 3 g/L compared to 2 g/L for Q-PAO1ΔaroA-RhlAB. This suggests that Shanghai-derived WFO is superior for rhamnolipid synthesis.

The acid value disparity (Q: 520 vs. S: 69 mg KOH/g) likely explains these differences. Q-WFO’s higher acid value (520 vs. 69) indicates severe lipid oxidation and free fatty acid (FFA) accumulation. High FFA levels can inhibit bacterial growth and metabolism, consistent with Q-WFO’s lower yields. In contrast, S-WFO’s lower acid value signifies superior lipid quality, enabling higher rhamnolipid production.

Strain performance differed by WFO source. PAO1-RhlAB showed a steeper increase in yield with S-WFO, reaching 10 g/L at 216 h, whereas PAO1ΔaroARhlAB exhibited slower growth but maintained stable production across both substrates. This suggests that RhlAB overexpression enhances adaptation to low-acid-value WFO, while *aroA* knockout strains are less sensitive to acid value differences.

In summary, waste oil origin and acid value critically determine rhamnolipid yields. Shanghai-derived WFO, with lower acid value, is a superior substrate. These findings highlight the importance of selecting high-quality waste oils for industrial rhamnolipid production and provide a basis for optimizing substrate pre-treatment processes (Onn et al., 2024).

### 3.3 Lipase enhances rhamnolipid production by PAO1 utilizing WFO

**Figure 3:**
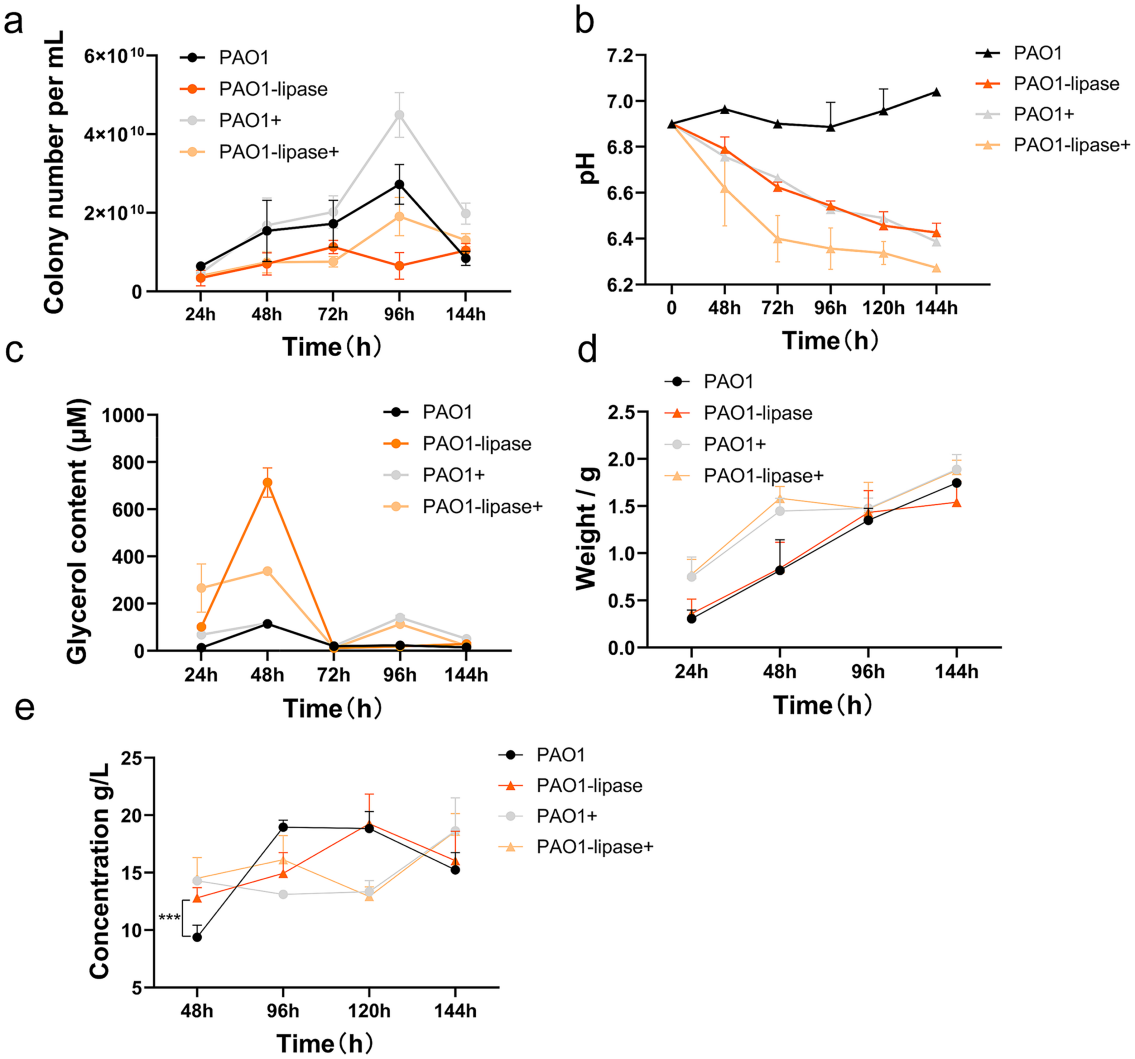
Effect of lipase on rhamnolipid production by *P. aeruginosa* utilizing WFO. **(a)** Growth curves (OD₆₀₀) of four *P. aeruginosa* systems: wild-type PAO1, PAO1-lipase (lipase-overexpressing), PAO1+ (wild-type with exogenous lipase), and PAO1-lipase+ (lipase-overexpressing with exogenous lipase). PAO1-lipase and PAO1-lipase+ show accelerated growth, indicating enhanced WFO utilization via lipase. **(b)** pH dynamics during fermentation. **(c)** The glycerol content in the fermentation broth. **(d)** Residual biomass (dry weight, g) over time. **(e)** Rhamnolipid concentration (g/L) profiles. Data are presented as means ± standard deviation (*n* = 3).

To investigate the effect of lipase on rhamnolipid production by *P. aeruginosa* PAO1 utilizing WFO (Shanghai), four systems were compared: wild-type PAO1, PAO1-lipase (lipase-overexpressing recombinant strain), PAO1+ (wild-type with exogenous lipase added at 0, 48, 96, 120, and 144 h, 40 U each), and PAO1-lipase+ (lipase-overexpressing strain with the same exogenous lipase addition as PAO1+). Growth curves, pH dynamics, glycerol content, oil degradation rate, and rhamnolipids yield were monitored over 144 h (Figure 3).

Early fermentation (48 h): Growth curves (Figure 3a) showed stable proliferation across strains, with PAO1-lipase and PAO1-lipase+ having slightly higher cell densities. pH (Figure 3b) remained stable (6.8–7.0), though PAO1+ and PAO1-lipase+ were marginally lower—likely due to exogenous lipase accelerating lipid hydrolysis and releasing free fatty acids (FFA). Glycerol (Figure 3c) was elevated in PAO1-lipase, PAO1-lipase+, and PAO1+, reflecting lipase-driven lipid breakdown. Oil degradation (Figure 3d) was highest in PAO1+ and PAO1-lipase+ (~24.80%), far exceeding PAO1 and PAO1-lipase (~13.60%). Rhamnolipid yields (Figure 3e) followed: PAO1-lipase+ (15 g/L) > PAO1+ (14 g/L) > PAO1-lipase (12 g/L) > PAO1 (9 g/L), confirming synergistic effects of overexpression and supplementation.

Mid-fermentation (96 h): Growth curves (Figure 3a) showed stable cell densities across groups. pH (Figure 3b) declined: PAO1 (6.8) > PAO1-lipase+ (6.7) > PAO1-lipase (6.5) > PAO1+ (6.4)—aligning with lipid hydrolysis rates (slower breakdown in PAO1 reduced FFA, preserving higher pH). Glycerol (Figure 3c) decreased, with PAO1+ showing the steepest drop (consistent with high degradation). Oil degradation (Figure 3d) remained highest in PAO1+ (27.1%), followed by PAO1-lipase+ (26.4%), PAO1-lipase (23.2%), and PAO1 (21.6%).

Rhamnolipid yields (Figure 3e) correlated with pH: PAO1 (19 g/L, pH 6.8) > PAO1-lipase+ (17 g/L, pH 6.7) > PAO1-lipase (16 g/L, pH 6.5) > PAO1+ (13.2 g/L, pH 6.4), indicating acidic conditions (excessive FFA) suppressed synthesis.

Late fermentation (144 h): Growth curves (Figure 3a) showed stable growth, with PAO1+ and PAO1-lipase+ having slightly higher densities. pH (Figure 3b) declined to 6.2–6.7: PAO1 (6.7) > PAO1-lipase+ (6.6) > PAO1-lipase (6.3) > PAO1+ (6.2). Glycerol (Figure 3c) was nearly depleted, reflecting maximal utilization. Oil degradation (Figure 3d) was highest in PAO1-lipase+ (34.40%). Rhamnolipid yields (Figure 3e): PAO1+ (20 g/L) > PAO1-lipase+ (19 g/L) > PAO1-lipase (17 g/L) > PAO1 (15 g/L), with PAO1+ balancing late-stage FFA metabolism effectively.

These results highlight that exogenous lipase addition offers simplicity and cost-effectiveness with strong early and late performance, while PAO1-lipase+ excels in the mid-stage through synergistic activity, collectively enhancing WFO valorization into rhamnolipids.

This study demonstrates that genetically engineered *P. aeruginosa* strains and lipase regulation strategies effectively enhance rhamnolipid production from WFO. Key findings highlight WFO as a viable carbon source, with RhlAB overexpression boosting yields and *aroA* knockout combined with RhlAB overexpression partially restoring productivity, particularly in WFO. Notably, lipase regulation strategies show significant potential: exogenous lipase addition (PAO1+) accelerates early-stage production (14.3 g/L at 48 h, about doubling wild-type yields), shortening fermentation time, while maintaining high late-stage yields (20.3 g/L at 144 h). The synergistic system (PAO1-lipase+) further optimizes early efficiency (16.0 g/L at 48 h) and achieves the highest oil degradation rate (34.40% at 144 h), balancing rapid initial synthesis with sustained late-stage performance. This observation aligns with industrial-scale findings in polyhydroxyalkanoate (PHA) production via lipid fermentation, where endogenous lipase overexpression in *Cupriavidus necator* H16 was shown to enhance the utilization efficiency of diverse lipid substrates (including food-grade palm oil and crude waste cooking oil), ultimately leading to improved PHA titers by facilitating more efficient hydrolysis and assimilation of hydrophobic lipid carbon sources(Jiang et al., 2025). WFO sources with lower acid values (e.g., Shanghai) support superior productivity, emphasizing substrate quality’s role.

While this study confirms WFO as a viable carbon source for rhamnolipid production and lipase regulation (endogenous overexpression/exogenous addition) enhances WFO utilization, key challenges persist. First, WFO quality variability (e.g., acid value differences between Shanghai and Qingdao WFO causing 2.5-fold yield gaps) lacks mitigation via practical pre-treatment or quality criteria. Second, lipase-fermentation trade-offs remain unaddressed: exogenous lipase triggers transient pH drops (to 6.2–6.4) suppressing mid-stage yields, and lipase addition timing/cost-effectiveness for scaling are unoptimized. Resolving these is critical for industrial application. Future work should focus on optimizing combined genetic modifications to strengthen early-stage acceleration and late-stage yield stability, refining lipase addition protocols for industrial scalability, and exploring WFO pre-treatment to mitigate acid value impacts, ultimately advancing efficient, cost-effective rhamnolipid production from waste lipids. *Pseudomonas aeruginosa* (Risk Group 2, opportunistic pathogen) poses safety/regulatory challenges for industrial rhamnolipids production. Our lipase-based strategy may mitigate this, with potential extension to safe strains (e.g., non-pathogenic *Pseudomonas putioda*) lacking lipase expression. *Pseudomonas aeruginosa* validated the strategy; extending to safe strains is a promising future direction.

## Data Availability

The original contributions presented in the study are included in the article/Supplementary material, further inquiries can be directed to the corresponding author/s.
