# Supplementary material for: Genetically engineered Pseudomonas aeruginosa with lipase regulation for production of rhamnolipids from waste frying oil
Source: Front Microbiol. 2025 Nov 20;16:1691217. doi: 10.3389/fmicb.2025.1691217 (PMC12676701; doi:10.3389/fmicb.2025.1691217)
Supplement: Supplementary file 1 [file Data_Sheet_1.docx]

Supplementary Material

**Supplementary Table 1.** Strains and plasmids in this study.

| **Strains** | **Genotype** | **Source** |
| --- | --- | --- |
| *P. aeruginosa* |  |  |
| PAO1 | Wild type | Our lab |
| PAO1ΔaroA  PAO1-RhlAB  PAO1ΔaroA-RhlAB  PAO1-lipase | PAO1, knock out the *aroA* gene (The *aroA* gene encodes the enzyme 5-enolpyruvylshikimate-3-phosphate synthase)  PAO1, pBBR1-RhlAB-estA-genta  PAO1ΔaroA, pBBR1-RhlAB-estA-genta  PAO1, pBBR1-lac-lipA-lipB-genta | Our lab  This study  This study  This study |
| **Plasmids** |  |  |
| pBBR1-RhlAB-estA-genta | pBBR1 replicon, *RhlAI*, *RhlB*, *estA*, *gentaR* | Our lab |
| pBBR1-lac-lipA-lipB-genta | pBBR1 replicon, *lacZ*, *lipA*, *lipB*, *gentaR* | Our lab |


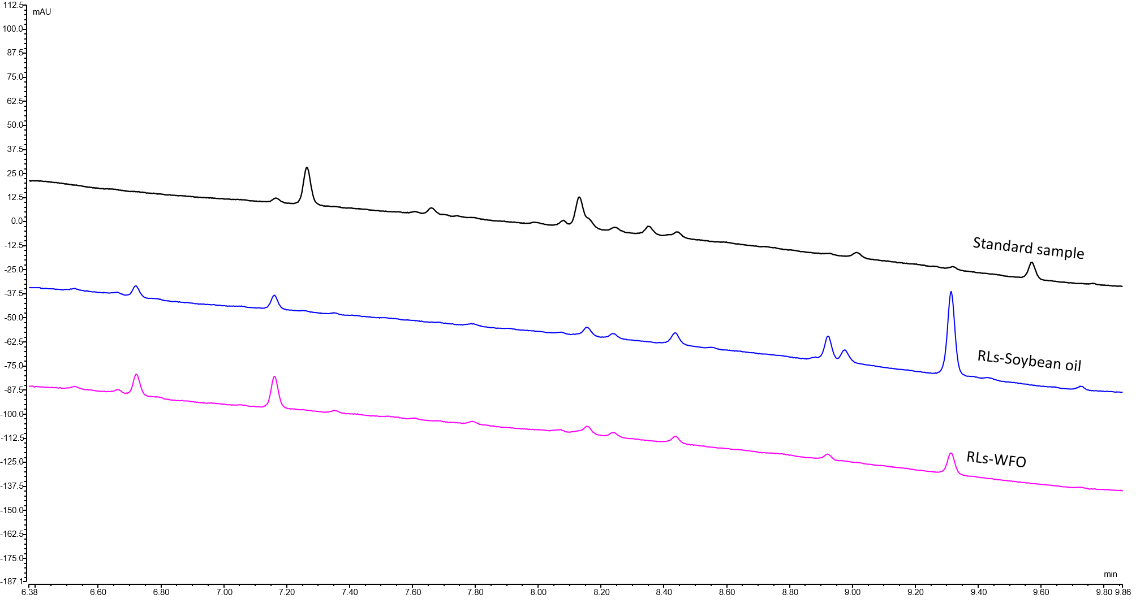


**Supplementary Figure 1.** UV spectrum of standard sample of rhamnolipids, rhamnolipids obtained by fermentation with soybean oil (RLs - Soybean oil), and rhamnolipids obtained by fermentation with waste frying oil (RLs - WFO). The figure presents a representative result, with the underlying experiment conducted in three biological replicates (n = 3) for reliability.


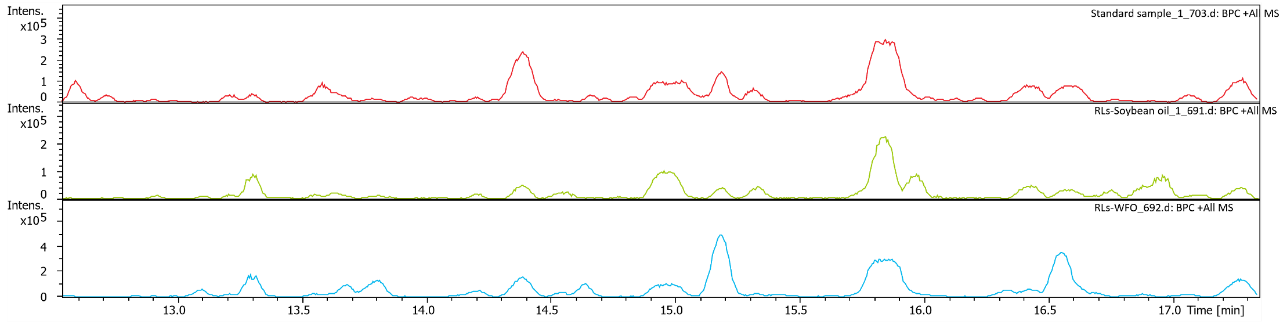


**Supplementary Figure 2.** MS BPC spectrum of standard sample of rhamnolipids, rhamnolipids obtained by fermentation with soybean oil (RLs - Soybean oil), and rhamnolipids obtained by fermentation with waste frying oil (RLs - WFO). The figure presents a representative result, with the underlying experiment conducted in three biological replicates (n = 3) for reliability.


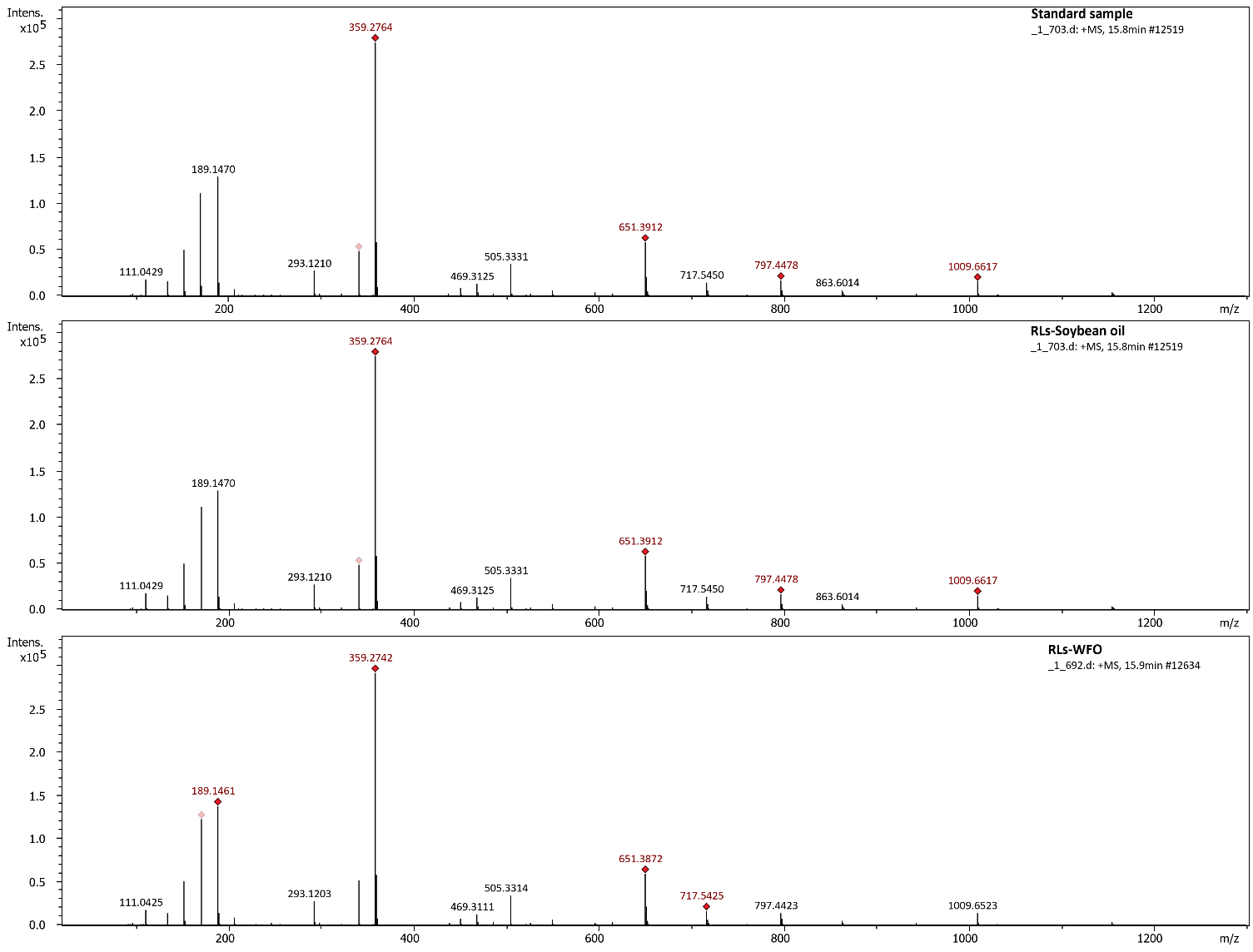


**Supplementary Figure 3.** MS/MS of rhamnolipids, standard sample of rhamnolipids, rhamnolipids obtained by fermentation with soybean oil (RLs - Soybean oil), and rhamnolipids obtained by fermentation with waste frying oil (RLs - WFO). The figure presents a representative result, with the underlying experiment conducted in three biological replicates (n = 3) for reliability.


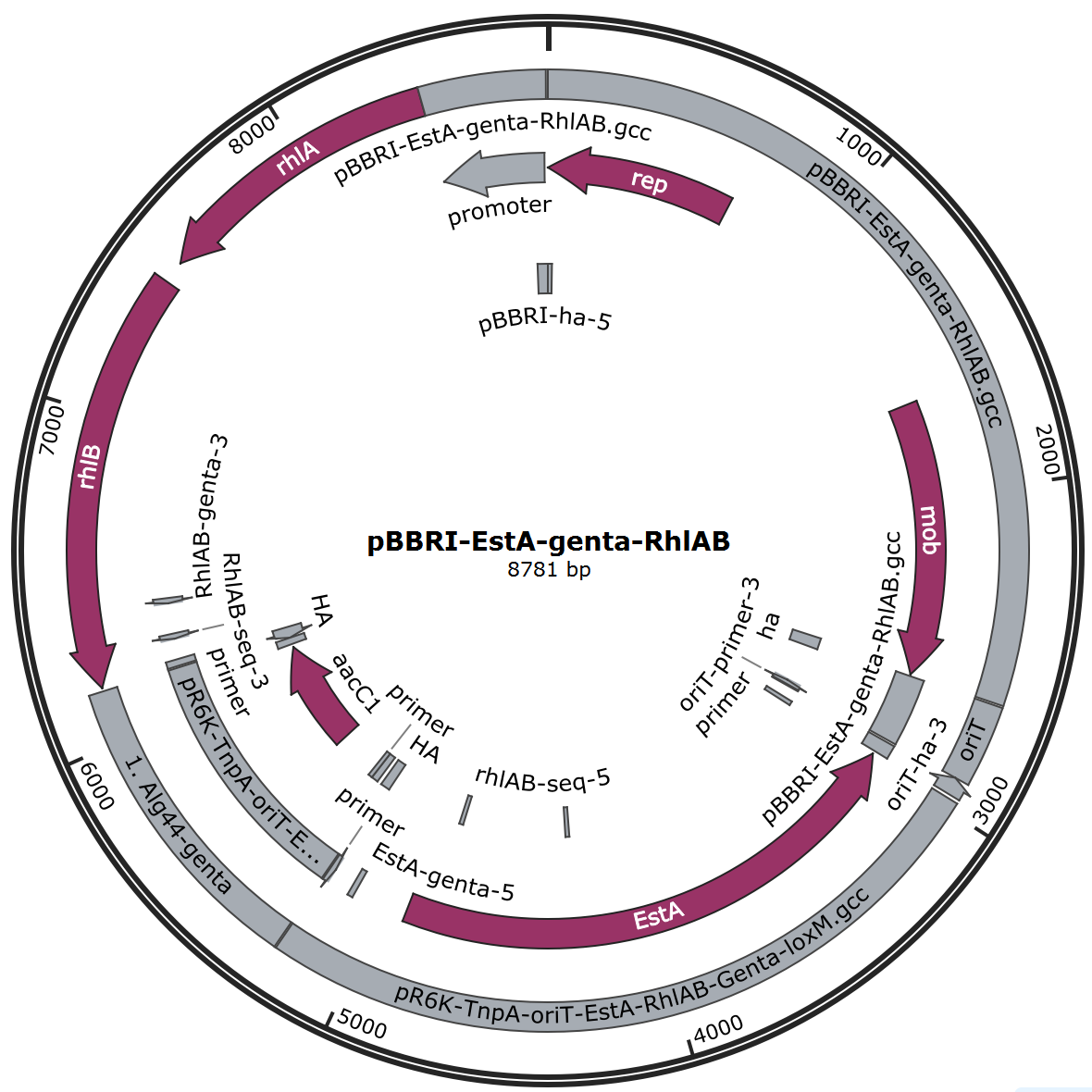


**Supplementary Figure 4.**  Schematic map of the plasmid pBBR1-RhlAB-estA-genta.


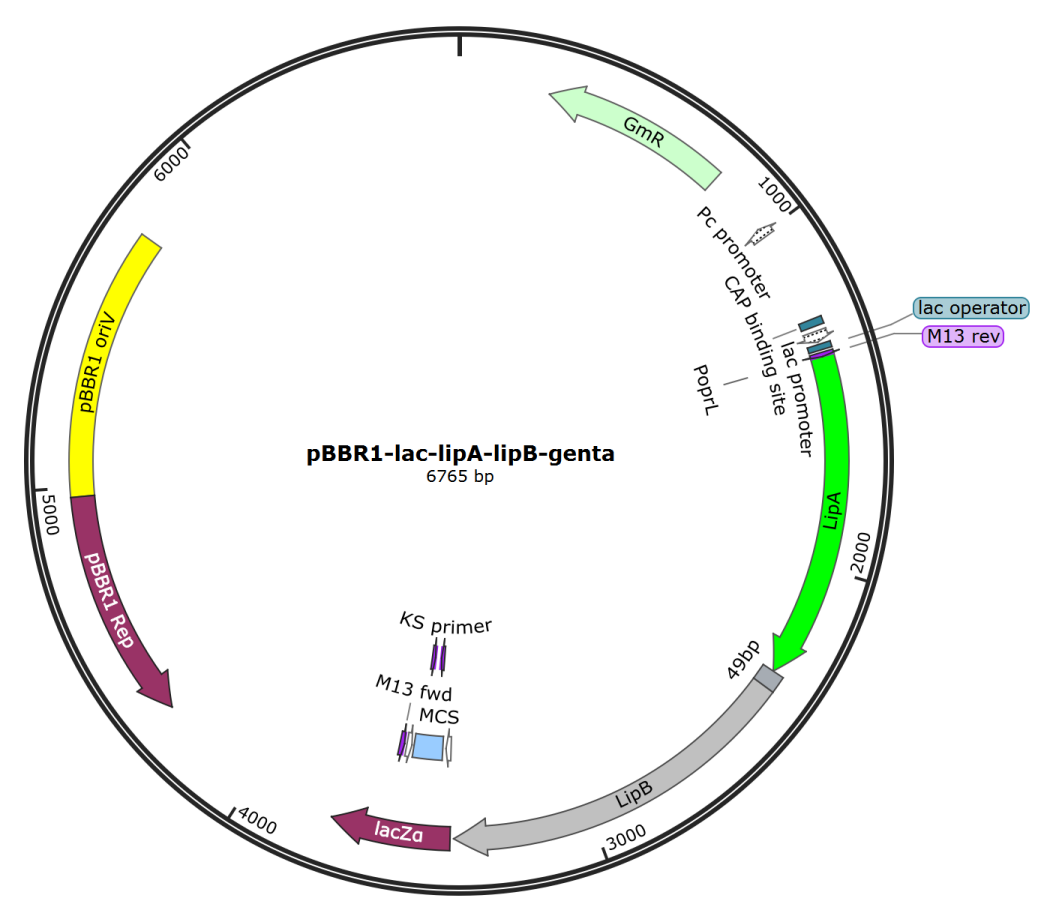


**Supplementary Figure 5.**  Schematic map of the plasmid pBBR1-lac-lipA-lipB-genta.
